# Barriers and attitudes towards cervical cancer screening in primary healthcare in Poland - doctors’ perspective

**DOI:** 10.1186/s12875-021-01612-8

**Published:** 2021-12-30

**Authors:** Katarzyna Nessler, Francis Ball, Sze Kay Florence Chan, Michal Chwalek, Anna Krztoń-Królewiecka, Adam Windak

**Affiliations:** 1 Department of Family Medicine, Jagiellonian University Medical College, Bocheńska 4, 31-061 Kraków, Poland; 2 Department of Family Medicine, Students’ Family Medicine Interest Group, Jagiellonian University Medical College, Kraków, Poland

**Keywords:** Cancer, Cervix, Cancer, Cancer epidemiology, General practice, Health behaviour, Preventive medicine

## Abstract

**Background:**

Healthcare systems have adopted different strategies to reduce the burden of cervical cancer. In Poland, a population-based screening program was implemented in 2006, leading to a downward trend in cervical cancer burden. However, screening rates are still low in relation to other EU member states. In Poland, Pap smears are mainly performed by gynecologists rather than Primary Health Care (PHC) physicians. Little is known about the experiences and attitudes of the latter regarding cervical cancer screening in a PHC setting.

**Methods:**

A cross-sectional questionnaire-based survey was carried out among 43 PHC physicians from the Malopolska region in Poland. Barriers and attitudes towards cytology in a PHC setting were evaluated.

**Results:**

Approximately 35% of surveyed physicians reported having experience in performing cytology. Almost 75% of PHC physicians lacked the necessary equipment in their office to perform the screening. None of the studied physicians performed Pap smears in their office at the time. The reasons included: shortage of competence (78.57%) and time (69.05%), the perception of Pap smears as a task for gynecologists (69.05%), the lack of financial incentives (61.90%), and the belief that their patients would be unwilling to undergo the test in their PHC physician’s office (33.33%). More than three quarters (76.74%) declared they would be ready to perform Pap smears if the tests were additionally paid. No significant associations between PHC physicians’ characteristics and their willingness to perform cytology screening were found.

**Conclusion:**

The primary barrier to perform Pap smears by PHC physicians does not lie in their personal reluctance but in the organization of the healthcare system. Provision of required training and proper funding allocation can likely improve the screening rate of cervical cancer in Poland.

## Introduction

Human papillomavirus (HPV) infection is the most common viral infection of the reproductive tract and persistent infection with high-risk HPV (hr-HPV) types is strongly associated with cervical cancer development [1–3]. Cervical cancer, the most common HPV-related cancer [4], is the fourth most frequent in terms of incidence and mortality in women worldwide [5] and the second most frequent cancer after breast cancer to affect women aged 15–44 years in the European Union [6].

In May 2018, the WHO Director-General made a call to action towards the elimination of cervical cancer and encouraged countries to increase access to, and coverage of, essential interventions to prevent the disease [7]. According to the European guidelines, co-testing (HPV and cytology primary testing) at any given age should be avoided, as only one test - either Pap smear or testing for oncogenic HPV should be used [8]. The Pap smear screening policy in Poland is in line with the recommended policies in the EU countries. Primary HPV testing with cytology triage (or nowadays possibly with other triage techniques) is an alternative for Pap smear testing.

Since implementing new vaccination regimens addresses only a part of the public health problem, a multidimensional approach including public education campaigns, training of health workers, and increased access to quality screening continues to be of utmost importance [1]. Healthcare systems around the world have faced different barriers to achieving these goals and addressed the problem variably. In Poland, a population-based screening program was implemented in 2006, which was soon observed to accelerate the downward trend in cervical cancer burden [9].

The population-based screening program for cervical cancer in Poland is fully covered by the National Health Fund (NHF). Every 3 years, all insured women aged 25–59 (~ 95% of women in this age group) are sent an invitation to have a Pap smear taken. The medical staff not only perform the test, but also inform women about the received results and refer for colposcopy and biopsy if needed. Unfortunately, the compliance to invitations is very low (approximately 10–13% between 2007 and 2013) [10].

Currently, Pap smear samples are frequently taken in gynecological outpatient clinics functioning mainly in the private sector, where women can make an appointment with a doctor or obstetrics nurse. Even though the national screening registry has been implemented, it is not fully integrated with other, mainly non-public services and in consequence much data is lacking. The total number of Pap smears collected in Poland outside the population-based screening program is unknown. Also, there is no registry of cervical histology results obtained outside the program. There is no automatic or obligatory reporting of histology results from the labs to a cancer registry. Moreover, HPV testing is not reimbursed within the program for triage of abnormal Pap results. Also, the percentage of women referred for colposcopy/biopsy who underwent colposcopy/biopsy within the program has been reported as low (approximately 30–50% between 2007 and 2013) [10].

This data shows that the population-based screening program for cervical cancer in Poland does not function properly and there are many shortcomings comparing to the European Guidelines for Quality Assurance in Cervical Cancer Screening [11, 12].

However, estimates for 2018 indicate that about 1900 new cervical cancer cases are still diagnosed annually [5].

In a recent study of 29 European countries, the cases where the general practitioner was not the Pap smear sample taker were very few [13]. The population coverage of the screening test in Poland stayed far behind most other European countries. Many countries achieved a test coverage of 70% or more (Denmark, England, Finland, Iceland, Ireland, Italy, Scotland, Slovenia, Sweden, and Wales), as compared to approximately 25% in Poland [13]. It was also estimated that 2/3 of all Pap smears in Poland were taken outside the nationally organized population-based screening program that is provided free of charge.

In Poland PHC physicians are allowed to perform Pap smears for screening and/or diagnostic reasons. Female patients visit their PHC doctors for urogenital symptoms or sexual problems, but very rarely have Pap smears taken in their offices.

Our study aims to answer the following questions:

1. What are the barriers and attitudes of Polish PHC physicians towards performing Pap smears in their offices?
2. Are there any associations between the demographic and professional characteristics of PHC physicians that could influence their readiness to perform Pap smears in their offices?

## Material and methods

### Study design

A cross-sectional questionnaire-based study was conducted from January to December 2018 in the Małopolska region (Krakow and the surrounding small towns and villages). The data was collected by medical students, who had all received appropriate training and instructions regarding the study protocol. This study is a part of a survey performed among PHC doctors and their patients. Detailed information about the study design was published elsewhere [14].

In brief, phone inquiries were made to the managers of randomly selected practices to ask for their permission to conduct the study in their office. Once they granted their consent, one physician from each practice was invited to participate in the study. On the set date, a fieldworker visited the participating PHC practices, obtained written consent from PHC physicians, and handed them the questionnaire. The purpose of the study was thoroughly explained to all participants, and a written informed consent was obtained from each respondent.

All physicians completed the survey and returned it to the students in the provided sealed envelopes.

The study was conducted according to GCP rules and confidentiality was maintained throughout. The survey received was approved by the Jagiellonian University Bioethics Committee’s decision no KBET/122.6120.15.2017 on January 26th, 2017.

### Sampling

The sample size was calculated using OpenEpi software with the following assumptions:

Number of primary care practices registered in Malopolska region 702 (N), a pilot study showed that 87% of physicians are willing to perform Pap smear in a PHC office (p) with confidence limits as % of 100(absolute +/− %) (d) = 10% and a design effect (DEFF) = 1. Using the equation Sample size n = [DEFF*Np(1-p)]/ [(d2/Z21-α/2*(N-1) + p*(1-p)], a sample size (n) of 41 physicians.

However, since physicians are recognized as a professional group from which feedback is normally difficult to obtain a final sample size of 200 practices was randomly selected to reach the minimum number of respondents. The study relied on simple random sampling by means of a random-number table to draw a sample from a local register of PHC physicians provided by the National Health Service Fund.

### Research tool

Based on an initial literature review a special questionnaire was designed for the purposes of the study [12, 13, 15–18]. The initial questionnaire was validated in a pilot study, where 10 doctors were invited to evaluate its face validity. The final version of the questionnaire was developed based on their comments. We then used Cronbach’s alpha to see if the multiple-item Likert scale questions were reliable. Acceptable reliability levels were achieved for each set (Cronbach’s alpha: 0.618; 0.642; 0.722).

The final questionnaire filled out by physicians consisted of 16 questions, of which 3 were multi-item questions with a 5-point Likert scale (1-strongly agree, 2-agree, 3-neutral, 4-disagree, 5-strongly disagree), 5 were semi-open, and the rest had predefined answers. Data was collected concerning the physicians’ sex, age, the location of their office, the number of patients they saw per week, their medical specialty, and the number of years in practice. The questionnaire also included questions about their experience in performing Pap smears, their reasons for not doing so in their PHC office, as well as their willingness to introduce the test in their practice. One question specifically examined their perception of the potential benefits of Pap smears in PHC; another addressed the possible obstacles to this procedure.

### Statistical analysis

Statistical analysis was conducted using Statistica 13.1 software (Dell Inc.) and descriptive statistics were used to present the results. To investigate the associations between the doctors’ characteristics and their attitude towards Pap smears in a PHC setting, the Chi-square test was used for qualitative and the Mann-Whitney U test for quantitative variables. An alpha level of *p* = 0.05 was considered statistically significant.

## Results

### Physicians’ characteristics

Out of the 200 primary care physicians we approached, 43 agreed to participate in the study (giving a response rate of 21.5%). Their mean age was 39.5 years (± SD 9.25). Most (79%) specialized in family medicine only, three (7%) specialized in internal medicine only, while 14% had two medical specialties: family medicine and internal medicine (7%) or pediatrics (7%). Almost half of the respondents (46%) practiced in a city of more than 100,000 inhabitants. The mean number of patient visits per week across all practices was 143 (± SD 75). The detailed characteristics of respondents are presented in Table 1.

**Table 1:** Characteristics of respondents

Feature	Total N (%)	Willing to perform Pap smear N (%)	Unwilling to perform Pap smear N (%)	*P*-value
**Gender**				
**Women**	24 (55.81)	16 (48.48)	8 (80.00)	0.079
**Men**	19 (44.19)	17 (51.52)	2 (20.00)	
**Experience in PC**				
**< 5 years**	12 (27.91)	11 (33.33)	1 (10.00)	0.305
**5–10 years**	16 (37.21)	12 (36.36)	4 (40.00)	
**> 10 years**	15 (34.88)	10 (30.30)	5 (50.00)	
**Specialty**				
**Family Medicine**	40 (93.02)	31 (93.94)	9 (90.00)	0.668
**Other**	3 (6.98)	2 (6.06)	1 (10.00)	
**Place of work**				
**Big city**	20 (46.51)	12 (36.36)	5 (50.00)	0.731
**Small town**	6 (13.95)	5 (15.15)	1 (10.00)	
**Village**	17 (39.53)	16 (48.48)	4 (40.00)	
**Patients’ structure**				
**Adults** **only**	11 (25.58)	25 (75.76)	7 (70.00)	0.714
**Adults and children**	32 (74.42)	8 (24.24)	3 (30.00)	
**Experience in pap smear performance**				
**Yes**	15 (34.88)	13 (39.39)	2 (20.00)	0.260
**No**	28 (65.12)	20 (60.61)	8 (80.00)	
**Median age (Q1;Q3)**	36 (32;48)	34 (30;47)	43 (35;50)	0.136
**Median number of patient visits per week (Q1;Q3)**	150 (100;200)	120 (82;155)	162 (150;200)	0.072

### PHC physicians’ experience with cytology testing

Approximately one in three physicians (34.88%) confirmed having experience in performing cytology. Only one quarter (25.58%) indicated that they had the equipment, gynecological chair, etc. to do so in their office. However, none of the studied physicians performed Pap smears in their office at the time of the study. The reasons included: the lack of proper training and skills (78.57%), the lack of time (69.05%), the perception of Pap smears as a task for gynecologists (69.05%), the lack of an additional fee for this service (61.90%), and the belief that patients would be unwilling to undergo the test in their PHC physician’s office (33.33%).

### Attitudes of PHC doctors’ towards performing pap smears in their offices

More than three quarters of all respondents (76.74%) declared they would be willing to perform Pap smears if the test was additionally paid. Respondents expected a fee that would cover at least the costs of the basic procedures.

**Fig. 1:**
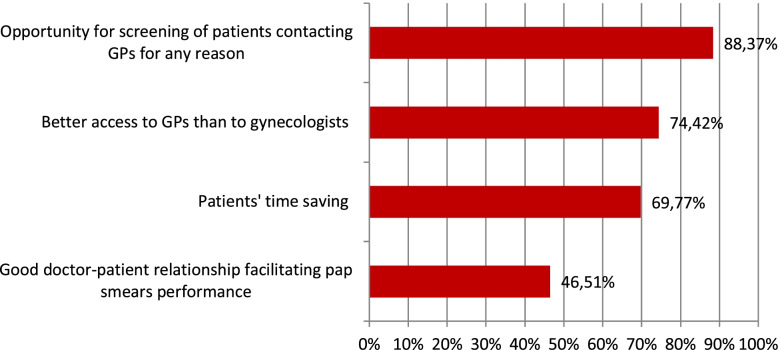
Advantages seen by PHC physicians if pap smears were available in PHC offices-results of predefined and open questions

The advantages of Pap smears being available in PHC offices, as seen by PHC physicians, are presented in Fig. 1.

**Fig. 2:**
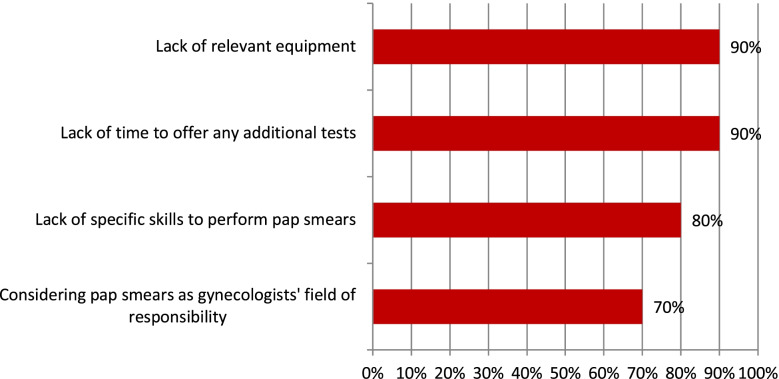
The reasons for not performing pap smears in PHC even if there was additional payment- results of predefined and open questions

The reasons why they would not perform Pap smears even if they were additionally paid are presented in Fig. 2.

### PHC physicians’ characteristics and their willingness to perform pap smears in their PHC office

We found no significant associations between the characteristics of our respondents and their willingness to perform cytology screening (Table 1). Furthermore, physicians’ age (*p* = 0.134) and workload, as defined by the number of patients visits per week (*p* = 0.060), were not found to be determining factors; however, workload approached the threshold of statistical significance.

## Discussion

### Summary of main results

The study revealed that none of the studied physicians currently performed Pap smears in their PHC office. The reasoning behind this was that they lacked the clinical experience and time to do so, and that cytology was considered to be a procedure reserved for gynecologists.

Only about one quarter indicated that they had the facilities and materials required to perform Pap smears in their office. Almost 90% of surveyed doctors believed that if the service was available at their office, they could promote and encourage Pap smear screening among their patients on different occasions.

Most respondents expected a fee that would cover at least the costs of the basic procedures.

Among physicians who were unwilling to perform Pap smears in a PHC setting, the main reasons were the lack of specialized equipment and too little time to offer additional services to their patients.

No significant association was found between any individual characteristics factor and the willingness to perform Pap smears.

### Strengths and limitations of the study

The data was collected from PHC physicians to examine potential barriers that limit patient access to cytology testing at their offices. The study was conducted at randomly chosen PHC offices, without selection bias.

The study was conducted with the minimal sample size and included respondents working in urban, suburban, and rural communities. The study was performed anonymously so we may suspect that doctors answered the questions truthfully.

The study, however, also had some limitations. The study has been conducted in one area of Poland (the Małopolska region) with relatively low number of participants and, therefore, a larger study covering other regions is needed for better representation and generalizability of the findings for the whole country. As predicted, the response rate was relatively low, although comparable to similar studies.

### Comparison with other studies

A study conducted in 2016 in France, another country where PHC physicians do not typically carry out Pap smears, showed that almost one in three PHC physicians never performed cervical cancer screening [19]. This is still more often than in our study, where none of the studied PHC doctors performed Pap smears in their office. The study by Poncet et al. further found that the likelihood of abstaining from cervical cancer screening was more pronounced in male PHC physicians and the youngest age group [19]. This trend was also supported in an article by Rochoy et al. in 2017. published in France, which demonstrated that 78.4% of female and only 45.7% of male PHC physicians were engaged in the performance of Pap-smears [20]. As explained by one systematic review, this may also be because female physicians are generally found to screen more patients than their male counterparts [21].

In our study, there was no significant difference between male and female PHC physicians in terms of their willingness to perform Pap smears.

A study performed in 2019 by Maj et al. found that PHC physicians working in countries with a low PHC-physician-density per inhabitant were more likely to perform cervical cancer screening [15]. Conversely, PHC physicians working in countries with easier access to gynecological care, as well as in areas with a mean income lower than the national average, were less likely to perform cervical cancer screening [15]. Although the urban versus rural setting of the PHC physician was considered in our study, no statistical difference was observed in that respect.

In a study conducted in France by Favre et al., cervical smears performed by PHC physicians led to increased screening participation rates [16]. In their evaluation of the independent PHC physicians’ characteristics that predicted participation rates in patients, multivariate analysis showed that the only significant characteristic was whether the doctor performed smears or not. Although the authors of that study did not directly evaluate the willingness of PHC physicians to perform Pap smears, their findings are in line with our own observation that no associations exist between demographic and professional characteristics of PHC physicians’ and their willingness to perform cytology screening. Moreover, Favre et al.’s findings support our assertion that promoting the provision of Pap smears by PHC physicians would lead to greater coverage [16].

In an interesting recent study from Norway, where Pap smears are routinely carried out by PHC physicians, Bringedal et al. examined doctors’ characteristics to determine whether and why doctors recommended disease-specific cancer screening to their patients [22]. The study revealed that PHC physicians adhered to cancer screening guidelines more in general and that they recommended cervical cancer screening at a significantly higher rate in comparison to other specialists. This trend suggests that focusing efforts on implementing a cervical cancer screening system specifically in the PHC setting may be an efficient way to boost patient awareness, accessibility, and adherence.

Most of the respondents in our study declared that if the service was available at their office, PHC physicians could promote and encourage Pap smear screening to their patients. It is worth noting that the main reasons brought up by those unwilling to perform Pap smears were the lack of specialized equipment and too little time to offer additional services. We may suspect that if these are provided, they might be more eager to change their attitude.

It has been shown that to encourage PHC physicians to support a national population-based screening program in primary care, several organizational issues must first be discussed, and the solutions must be implemented [23].

### Implications for practice and screening policy

Promoting cervical cancer screening in a PHC setting might be an important strategy to increase overall screening rates. More than three quarters of physicians in our study are willing to provide cytology screening services to their patients. The barriers to performing Pap smears in a PHC setting are primarily of organizational kind and could be easily addressed. In addition, raising the awareness of the fact that in many countries Pap smears are mostly performed by family physicians as a routine procedure can help encourage Polish physicians to include them in their practice. The logistical barriers can be eliminated with proper funding by designated public health authorities [24]. The study establishes a baseline for additional public health funding to be allocated for preventive measures taken at PHC offices.

Furthermore, physicians’ beliefs may in turn positively influence their patients’ willingness to undergo screening [25, 26], which can help improve the overall screening rate from the standpoint of public health [27]. There is also a strong consensus that long waiting times for an appointment with a specialist are a huge barrier to cervical cancer screening [28]. Performing Pap smears in a PHC setting can alleviate the long waiting times at gynecology clinics and show that visiting PHC physicians for Pap smear purposes can be time-saving and convenient for patients [29]. Furthermore, in conjunction with the test, PHC offices can also offer public education to patients who may lack knowledge about cervical cancer and the need for screening [30]. Taken together, these factors are crucial for increasing cervical cancer screening rates in Poland, as greater accessibility makes it more likely that patients will accept and pursue screening procedures.

It has been shown that organized, population-based screening programs have the potential to achieve more efficient resource use and greater equity in systematically reaching the target population [13]. Giving Finland as an example, the attendance rate per invitation is 73%. The population-based screening program has markedly affected the cervical cancer rates in this country, where there has been an approximately 80% decrease in age-adjusted cervical cancer incidence and mortality rates since it was implemented [31].

In our study, the percentage of doctors who would be willing to perform Pap smears in their offices is high. There are many GP offices in Małopolska, also covering areas where gynecologists are not easily available. GPs collaborate closely with community midwives, who are their team members. Even though currently in Poland, certified community midwives, who are working in many GP offices are also eligible to collect Pap smears at GPs’ practices, it is performed very rarely. It seems that community midwives could easily be trained to support GPs in the implementation of the cervical cancer screening program, and even collect Pap smears themselves in collaboration and under the supervision of PHC physicians. To implement these changes, first appropriate research, piloting, and demonstration projects are necessary. However, the results of this study are promising and draw inferences for the development and implementation of a more effective cervical cancer screening policy in Poland.

## Conclusion

Pap smears are routinely carried out by PHC physicians in many countries worldwide; however, this is not a common practice in the Polish health care system. With cervical cancer rates still relatively high in Poland, it is crucial that changes are introduced to make Pap smear testing more accessible to the general population.

Our findings provide greater insight into the attitudes of physicians towards cervical cancer screening.

The results of our study indicate that most PHC physicians would be willing to perform Pap smears in their practice. Providing required training for PHC staff and proper funding allocation could likely contribute to introducing Pap smears into primary care in Poland.

## Data Availability

Data from the whole survey are available on request. Please contact the corresponding author: Dr. Katarzyna Nessler; Jagiellonian University Medical College, Department of Family Medicine; 31–061 Kraków, Bocheńska 4, Poland. Tel. + 48 12 430 55 93, Fax. + 48 12 430 55 84. katarzynanessler@gmail.com
